# Altered pattern of monocyte differentiation and monocyte-derived TGF-β1 in severe asthma

**DOI:** 10.1038/s41598-017-19105-z

**Published:** 2018-01-17

**Authors:** Chih-Hsing Hung, Chin-Chou Wang, Jau-Ling Suen, Chau-Chyun Sheu, Chang-Hung Kuo, Wei-Ting Liao, Yi-Hsin Yang, Chao-Chien Wu, Sum-Yee Leung, Ruay-Sheng Lai, Chi-Cheng Lin, Yu-Feng Wei, Chong-Yeh Lee, Ming-Shyan Huang, Shau-Ku Huang

**Affiliations:** 1 Department of Pediatrics, Kaohsiung Medical University Hospital, Kaohsiung Medical University, Kaohsiung, Taiwan; 2 Department of Pediatrics, Faculty of Pediatrics, College of Medicine, Kaohsiung Medical University, Kaohsiung, Taiwan; 3 Graduate Institute of Medicine, College of Medicine, Kaohsiung Medical University, Kaohsiung, Taiwan; 4 Department of Pediatrics, Kaohsiung Municipal Hsiao-Kang Hospital, Kaohsiung, Taiwan; 5 Research Center for Environmental Medicine, Kaohsiung Medical University, Kaohsiung, Taiwan; 6 Division of Pulmonary and Critical Care Medicine, Department of Internal Medicine, Kaohsiung Chang Gung Memorial Hospital and College of Medicine, Chang Gung University, Kaohsiung, Taiwan; 7 Department of Public Health, Kaohsiung Medical University, Kaohsiung, Taiwan; 8 Department of Medical Research, Kaohsiung Medical University Hospital, Kaohsiung, Taiwan; 9 Division of Pulmonary and Critical Care Medicine, Kaohsiung Medical University Hospital, Kaohsiung, Taiwan; 10 Department of Pediatrics, Kaohsiung Municipal Ta-Tung Hospital, Kaohsiung, Taiwan; 11 Department of Biotechnology, College of Life Science, Kaohsiung Medical University, Kaohsiung, Taiwan; 12 School of Pharmacy, Kaohsiung Medical University, Kaohsiung, Taiwan; 13 Division of Chest Medicine, Kaohsiung Veterans General Hospital, Kaohsiung, Kaohsiung, Taiwan; 14 Chest Division, Department of Internal Medicine, Antai Medical Care Cooperation, Antai Tian-Sheng Memorial Hospital, Pingtung, Taiwan; 15 Division of Chest Medicine, Department of Internal Medicine, E-Da Hospital, I-Shou University, Kaohsiung, Taiwan; 16 Cancer Center, Kaohsiung Medical University Hospital, Kaohsiung, Taiwan; 17 National Health Research Institutes, Miaoli County, Taiwan; 18 Johns Hopkins University School of Medicine, Baltimore, MD USA

## Abstract

CD14^+^ monocytes contain precursors for macrophages and fibrocytes, known to be involved in regulating airway remodeling in human asthma and distinguishable by the PM-2K marker. We sought to identify circulating subsets of PM-2K^+^ macrophage-like cells and evaluate their relationships to lung function, severity and control status. Circulating PM-2K^+^ macrophage-like cells and fibrocytes could be identified and distinguished between normal individuals (*N* = 152) and asthmatic subjects (*N* = 133) using multi-parametric flow cytometry. PM-2K^+^ macrophage-like cells were found to be significantly lower in asthmatic subjects, particularly noted for the CD14^−^PM-2K^+^ subset and PM-2K^+^CCR7^−^CD86^+^ cells in subjects with poor lung function (FEV%/FVC% < 80%) as compared to those of normal subjects and asthmatics with normal lung function, whereas the frequency of fibrocytes was higher in asthmatics and the CCR7^−^CD86^+^ subset distribution was significantly different in subjects with varying severity. Moreover, exogenous transforming growth factor beta 1 (TGF-β1) was found to inhibit the generation of PM-2K^+^ macrophage^-^like cells, but promote the growth of fibrocytes, from CD14^+^ monocytes^,^ and monocyte-derived TGF-β1 was found to correlate with the lung function, severity and control status in asthmatic patients. Collectively, aberrant differentiation of monocytes into PM-2K^+^ macrophage-like cell subsets and fibrocytes, together with increased monocyte-derived TGF-β1, characterized patients with severe asthma.

## Introduction

Asthma, a common and often debilitating disease, still remains a critical public health, medical and economical problem, particularly for the severe and persistent cases, where airway flow obstruction is often irreversible and is frequently not well controlled by intensive guideline-based therapy^1^. Currently, the exact mechanisms underlying the disease progression remains unclear.

Macrophages, fibrocytes and fibroblasts are known to be important in fibrogenesis and airway remodeling^2–4^, although their mechanisms of action remain to be defined. Lung resident macrophages mediate immune surveillance for inhaled pathogens or allergens in steady state; however, macrophages also contribute to airway remodeling in the context of chronic inflammation^5^. It has been demonstrated that tissue macrophages contains various populations of both local self-renewing and blood monocyte-derived macrophages^6^. Fibrocytes, which appear to be differentiated from circulating CD14^+^ monocytes, have been recognized as a potential source of tissue fibroblasts and mediating fibrotic response^7–9^, while TGF-β1 is known to be able to promote the growth and differentiation of fibrocytes^10^. However, the relative roles of macrophages and fibrocytes in asthma remain to be studied due to the lack of specific markers in these two heterogeneous populations^11^.

In the standard conditions of the culture of human macrophages, PM-2K is an established marker to identify macrophages and distinguish macrophages from fibrocytes in monocyte-derived cell populations^12^. In human lung biopsy samples, alveolar macrophages are also PM-2K-positive cells^13^. On the other hand, circulating fibrocytes express CD45 and collagen I. Therefore, we set up a multi-parametric panel using flow cytometry to determine whether PM-2K^+^ cells could be identified in circulation and evaluate their distributions in a case-control population. This study presents a novel finding that as compared to those in healthy controls, significantly lower frequency of circulating PM-2K^+^ macrophage-like cell subsets, but higher level of fibrocytes, was noted in subjects with asthma, which was associated with elevated monocyte-derived TGF-β1, correlating with severity and poor lung function.

## Results

### Identification of circulating fibrocytes and PM-2K^+^ cells in peripheral blood

**Figure 1:**
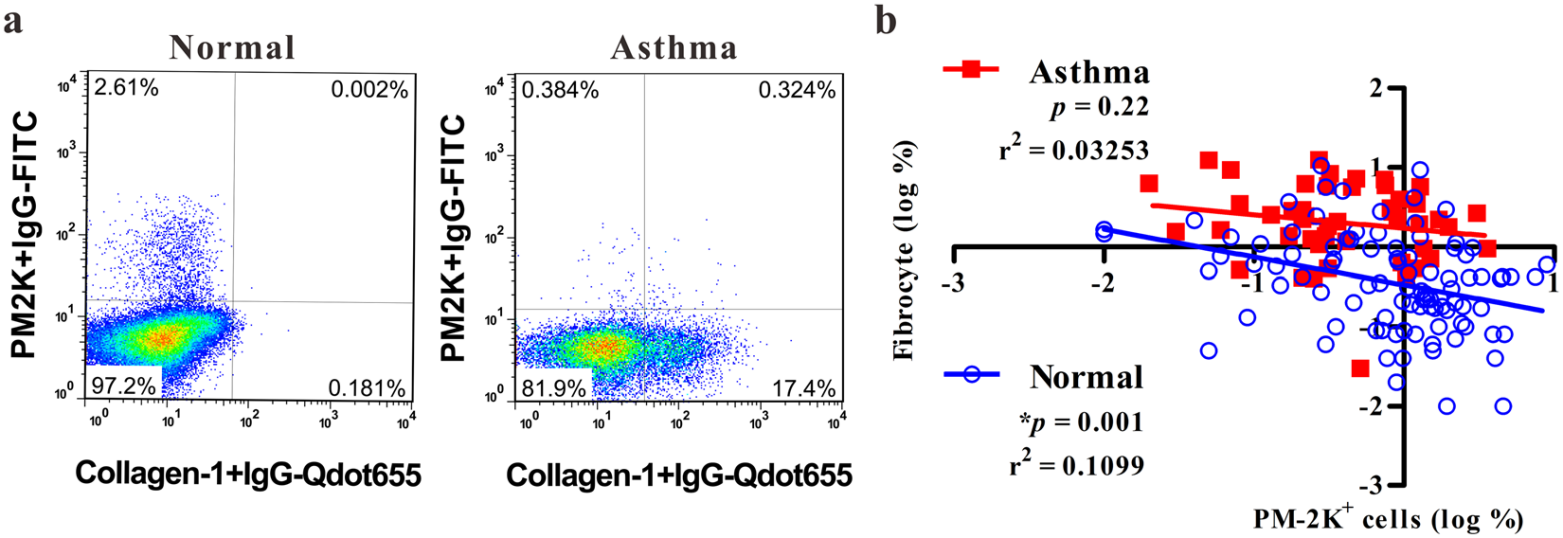
Analysis of circulating fibrocytes and PM-2K^+^ cells in peripheral blood mononuclear cells. (**a**) Representative dot plots of the respective dataset from normal and asthmatic patient. The intracellular collagen I^+^ (fibrocytes) and PM-2K^+^ cells are gated from viable CD3^−^CD19^−^CD45^+^ cells. (**b**) Their relationship was analyzed by linear regression based on the logarithm of percentages of fibrocytes or PM-2K^+^ cells in each individual. Normal, *N* = 92, Asthma, *N* = 48.

**Figure 2:**
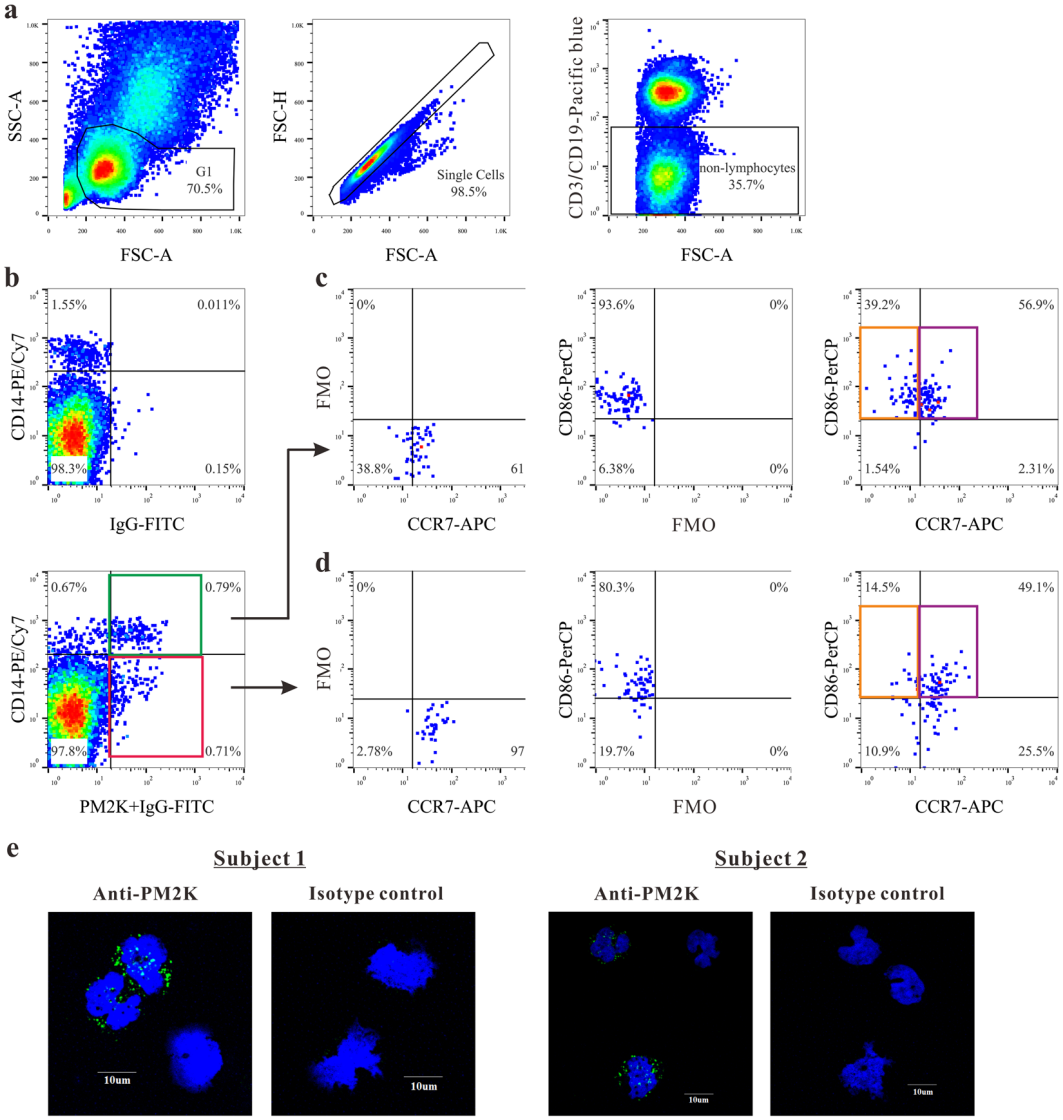
Identification of circulating PM-2K^+^ cells and its subsets. (**a**) The sequential gating strategy was shown on live and small cells (left), single cells (middle) and non-T/non-B cells (right). (**b**–**d**) PM-2K^+^ cells were gated against fluorescence minus one (FMO) control to identify the CCR7^+^CD86^+^ (purple gate) and CCR7^−^CD86^+^ (orange gate) subsets in PM-2K^+^CD14^+^ (green gate) and PM-2K^+^CD14^−^ (red gate) cells, using appropriate FMO controls. (**e**) Confocal imaging analysis of CD14^+^ cells from two normal subjects; PM-2K (green) and nucleus (blue).

As PM-2K marker has been used to differentiate *in vitro* monocyte-derived macrophages from fibrocytes^12^, we investigated whether circulating PM-2K^+^ cells and fibrocytes could be identified in the peripheral blood using a multi-color flow cytometry. We observed the existence of circulating PM-2K^+^ cells, designated as macrophage-like cells, and PM-2K^−^collagen I^+^ (fibrocytes) in normal controls and asthmatic patients (Fig. 1a). These circulating PM-2K^+^ cells were analyzed among the viable CD3^-^CD19^-^CD45^+^ cells as the gating strategy similar to Fig. 2a. In a case-control study population, in which no significant difference was found in age and gender distribution, except for the education level (Table 1), the relationship between circulating PM-2K^+^ cells and fibrocytes appeared to be in an inverse relationship in the normal control group, but not in the patient group (Fig. 1b). Consistent with a previous report^14^, a significantly higher level of fibrocytes was noted in subjects with asthma as compared to that of normal controls (Supplementary Fig. S1).

**Table 1:** Characteristics of the study population.

	Control *N* = 152	Asthma Case *N* = 133	*P* value
Gender *N* (%)			
Male	68 (44.7%)	58 (43.6%)	0.47
Female	84 (55.3%)	75 (56.4%)	
Age *N* (%)			
mean year (s.d.)	58.7 (11.8)	56.1 (16.5)	0.48
Below 54 year old	60 (39.5%)	52 (39.1%)	0.99
55–69 years old	59 (38.8%)	52 (39.1%)	
More than 70 years old	33 (21.7%)	29 (21.8%)	
Education Level *N* (%)			
Below junior high school	50 (32.9%)	66 (49.6%)	0.01*
Senior high School	60 (39.5%)	43 (32.3%)	
above bachelor	42 (27.6%)	24 (18.0%)	
Race *N* (%)			
Taiwanese	125 (82.2%)	109 (82.0%)	0.24
Hakka	7 (4.6%)	8 (6.0%)	
Born in China	18 (11.8%)	10 (7.5%)	
Aboriginal	2 (1.3%)	6 (4.5%)	
Smoking habits *N* (%)			
Never-smokers	122 (80.3%)	97 (72.9%)	0.34
Ex-smokers	17 (11.2%)	21 (15.8%)	
Smokers	13 (8.6%)	15 (11.3%)	
Passive smoking exposure at home *N* (%)			
No	51 (33.6%)	53 (39.8%)	0.16
Yes	101 (66.4%)	80 (60.2%)	
Passive smoking exposure at work *N* (%)			
No	108 (71.1%)	86 (64.7%)	0.15
Yes	44 (28.9%)	47 (35.3%)	

*P < 0.05.

### Phenotypic characterization of circulating PM-2K^+^ subsets in peripheral blood mononuclear cells (PBMCs)

To examine the heterogeneity of circulating PM-2K^+^ cells, three additional markers were chosen to characterize the PM-2K^+^ cells, including CD14 (monocyte/macrophage axis), CD86 (maturation marker), and CCR7 (homing receptor). As shown in Fig. 2a and b, we observed that PM-2K^+^ cells could be divided into two subsets based on their CD14 expression, which was defined as PM-2K^+^CD14^+^ and PM-2K^+^CD14^−^ subsets. Further, these two subsets were gated against fluorescence minus one (FMO) control to identify the CCR7^+^CD86^+^ and CCR7^−^CD86^+^ subsets in either the PM-2K^+^CD14^+^ (Fig. 2c) or PM-2K^+^CD14^−^ (Fig. 2d) cell populations. This was confirmed by confocal imaging analysis showing the existence of PM-2K^+^ cells in purified CD14^+^ cells from two normal subjects (Fig. 2e).

### Decreased percentage of PM-2K^+^ cells in peripheral blood of subjects with asthma

**Figure 3:**
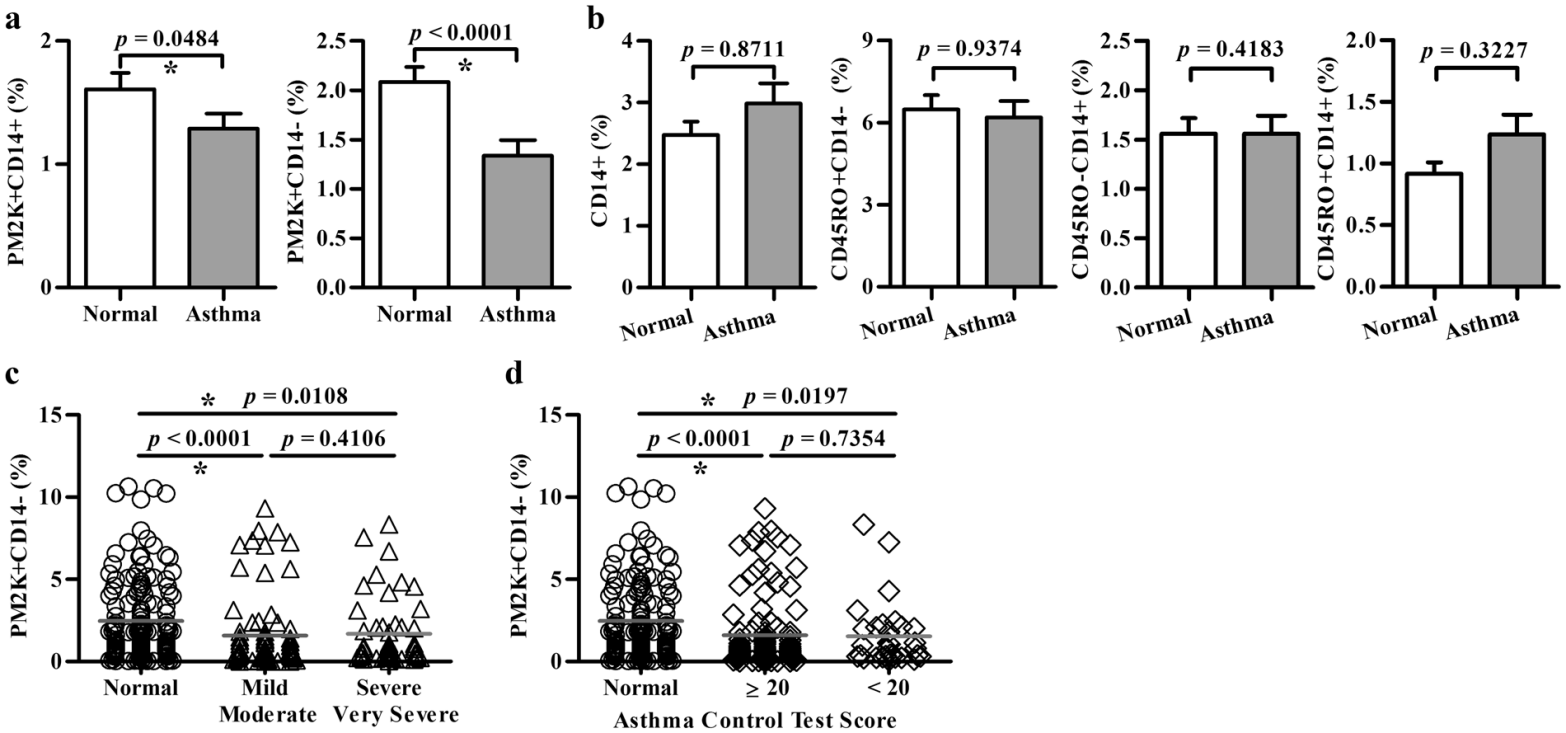
Analysis of circulating PM-2K^+^ subsets in a case-control study. The frequency (mean ± SEM) of various cell populations in viable CD45^+^ non-T/non-B cells in normal individuals (○) and patients (**a**, **b**) with varying severity (△) (**c**) and asthma control test score (◇) (**d**). Normal, *N* = 152, Asthma, *N* = 133. *P < 0.05 was considered significant.

Next, we examined the percentages of PM-2K^+^ subsets in the case-control population. The percentages of PM2K^+^CD14^+^ and PM2K^+^CD14^−^ cells were found to be significantly lower in subjects with asthma (mean ± SEM; 1.3 ± 0.1% and 1.3 ± 0.2%, respectively), when compared to those of normal healthy controls (1.6 ± 0.1% and 2.1 ± 0.2%, respectively) (Fig. 3a). The difference was more pronounced for the PM2K^+^CD14^-^ group (*P* < 0.0001). There was no significant difference in the percentages of CD14^+^ monocytes, CD45RO^+^CD14^−^, CD45RO^−^CD14^+^ and CD45RO^+^CD14^+^ subsets between the cases and the controls (Fig. 3b). Further, no significant difference in the frequency of PM2K^+^CD14^+^ or PM2K^+^CD14^−^ subset was found between mild/moderate and severe/very severe groups (Fig. 3c), or between well-control [asthma control test (ACT) score ≥20] and poor-control (ACT score < 20) cases (Fig. 3d).

### Altered distribution of circulating PM-2K^+^ subsets was associated with control status and lung function in asthmatic patients

**Figure 4:**
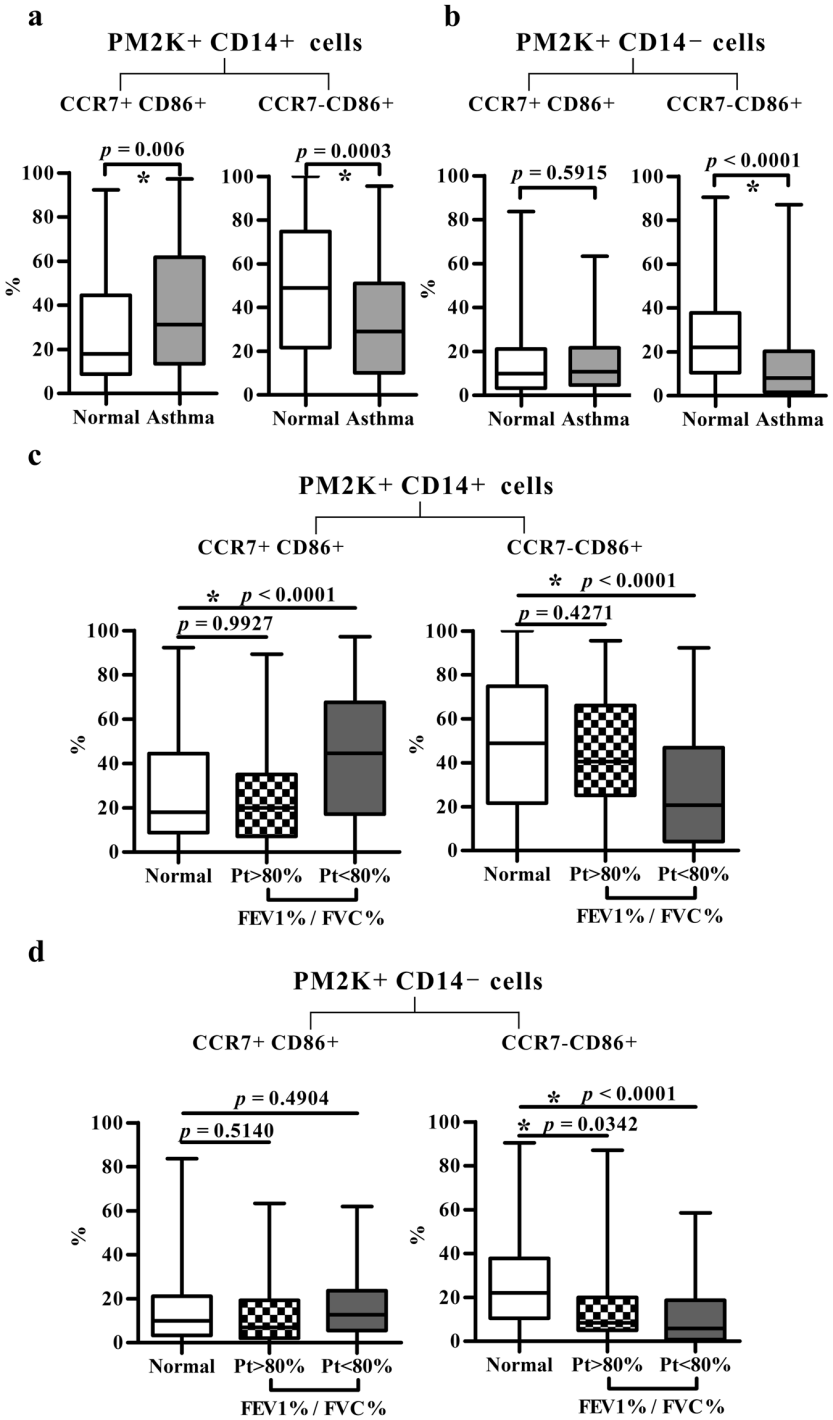
The distribution of PM-2K^+^ subsets. (**a**,**b**) CCR7^+^CD86^+^ and CCR7^−^CD86^+^ subsets in PM-2K^+^CD14^+^ and PM-2K^+^CD14^−^ subsets. Normal, *N* = 152, Asthma, *N* = 133. (**c**,**d**) CCR7^+^CD86^+^ and CCR7^−^CD86^+^ subset distribution in PM-2K^+^CD14^+^ and PM-2K^+^CD14^−^ subsets among normal individuals and patients (Pt) with different FEV1%/FVC% ratios (>80%, *N* = 28, <80%, *N* = 58); Normal, *N* = 152. Data are presented as box and whisker plots: boxes extend from the 25th to 75th percentiles, with a black line at the median; whiskers extend to show the highest and lowest values. *P < 0.05 was considered significant.

When the subsets of PM-2K^+^CD14^+^ and PM-2K^+^CD14^−^ were stratified by the expression of CCR7 and CD86, the frequency of the CCR7^+^CD86^+^ subset in asthmatic cases was significantly higher in the PM2K^+^CD14^+^ cell population (*P* = 0.006 versus controls) (Fig. 4a, left panel), but not in the PM-2K^+^CD14^−^ sub-population (Fig. 4b, left panel). The percentage of CCR7^−^CD86^+^ subset in both PM-2K^+^CD14^+^ (*P* = 0.0003) and PM-2K^+^CD14^−^ (*P* < 0.0001) cells were significantly lower in asthmatics as compared with normal controls (Fig. 4a and b, right panels).

Next, we examined the relationship between different PM-2K^+^ subsets and lung function. The percentage of CCR7^+^CD86^+^ subset in the PM-2K^+^CD14^+^ cells, but not the PM-2K^+^CD14^−^ cells, was significantly higher in asthmatic patients with poor lung function (FEV1%/FVC% < 80% of predicted value) (*P* < 0.0001) as compared with those of normal subjects and patients with poor lung function (Fig. 4c and d, left panels). In contrast, the percentage of CCR7^−^CD86^+^ subset in the PM-2K^+^ cells of both CD14^+^ and CD14^−^ populations was significantly lower, particularly in subjects with poor lung function when compared with normal controls (*P* < 0.0001) (Fig. 4c and d, right panels). Further, the frequencies of CCR7^−^CD86^+^ cells in both PM-2K^+^CD14^+^ and PM-2K^+^CD14^−^ subsets were statistically different between groups of mild/moderate and severe/very severe asthmatic subjects (Table 2), after correcting for multiple comparisons by Bonferroni correction. In addition, ROC analysis by computing the AUCs based on these two original measurements showed significance in their sensitivity and specificity in distinguishing the combined severe and very severe group of asthmatic subjects (Supplementary Table S1). These results suggested that the patterns of PM-2K^+^ subsets in the peripheral blood were significantly associated with patients’ severity and pulmonary function.

**Table 2:** The percentage of the CCR7^−^CD86^+^ subset is significantly different in subjects with varying severity.

Variable^1^	Mild/Moderate Group (*N* = 77) (Mean ± s.d.)	Severe/Very Severe Group (*N* = 53) (Mean ± s.d.)	*P* value
CCR7^−^CD86^+^ (%) in PM-2K^+^CD14^+^	28.3 ± 27.2	44 ± 25.7	*0.009
CCR7^−^CD86^+^ (%) in PM-2K^+^CD14^-^	9.9 ± 13.7	19.8 ± 20.3	*0.007

^1^Values are percentages of CCR7^−^CD86^+^ subsets in PM-2K^+^CD14^+^ or PM-2K^+^CD14^−^ non-T/non-B PBMCs.

*P < 0.05 was considered as significant.

### TGF-β1 suppressed the development of monocyte-derived PM-2K^+^ cells

**Figure 5:**
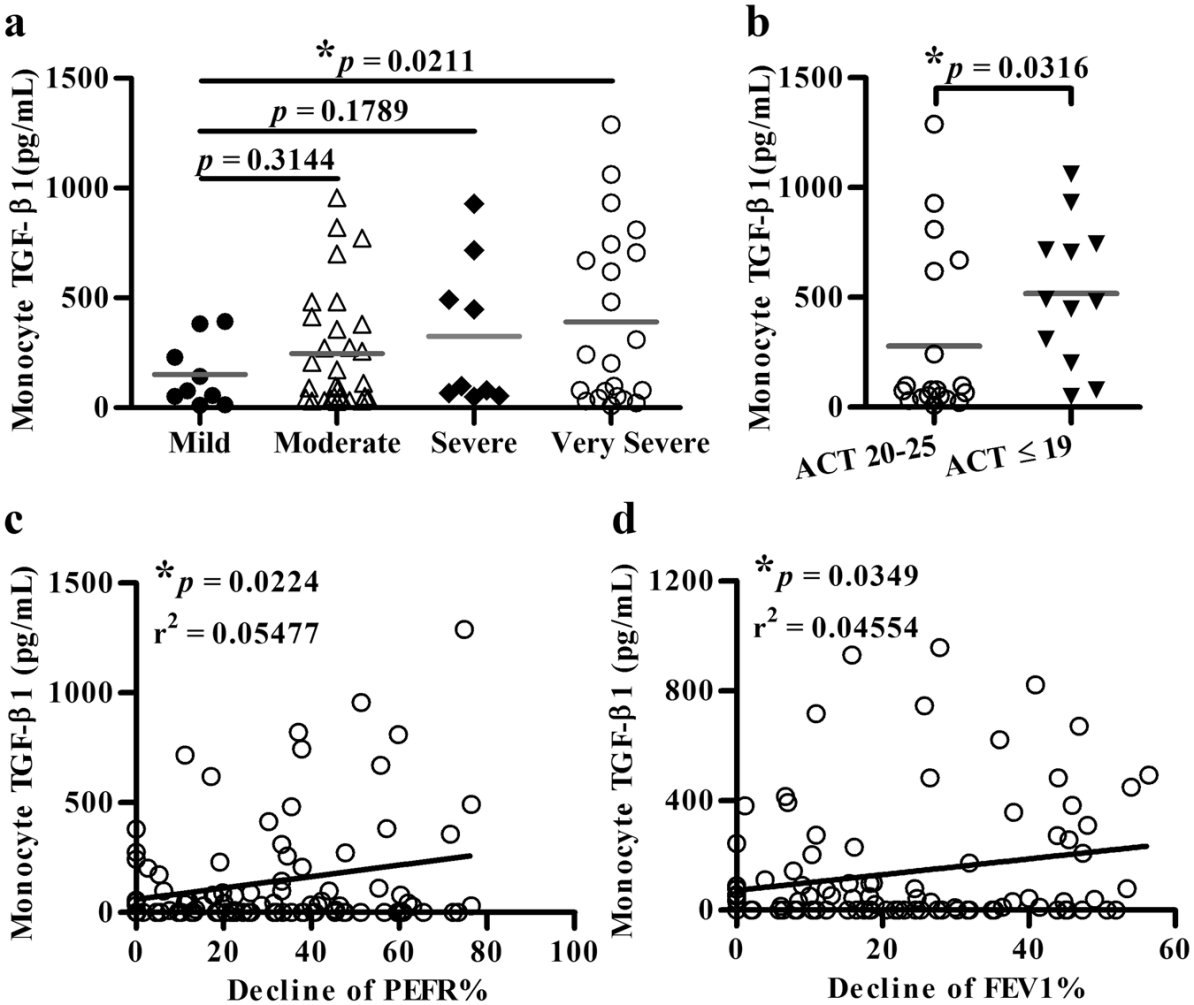
Analysis of TGF-β1 levels. Monocyte-derived TGF-β1 in patients with varying severity (**a**), ACT score (**b**), and their relationship with the decline of PEFR% (**c**) or of FEV1% (**d**).

To identify the likely mediators responsible for the change in monocyte differentiation, a panel of monocyte-derived cytokines and chemokines was analyzed. The results showed that the TGF-β1 levels were significantly higher in the patient versus the control group (Supplementary Table S2), which were particularly noted in very severe (Fig. 5a) and low ACT score (≤19) patient groups (Fig. 5b), and was found to be significantly associated with the decline of PEFR% (Fig. 5c) and of FEV1% (Fig. 5d) in the patient group.

**Figure 6:**
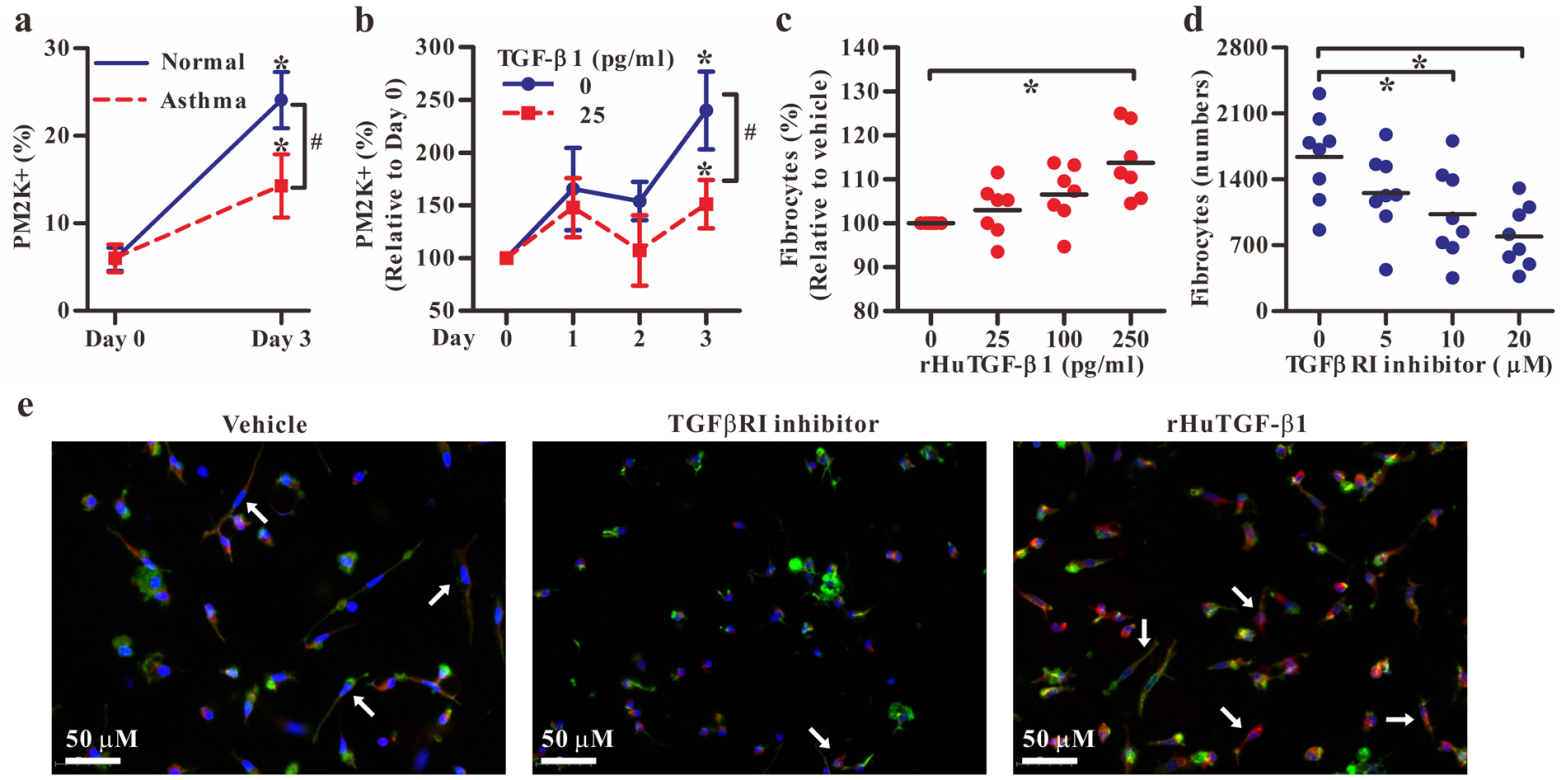
Effects of TGF-β1 on monocyte differentiation. (**a**) The percentages of PM-2K^+^ cells in CD14^+^ monocyte culture from normal individuals or patients. (mean ± SEM; *N* = 7/group) (**b**) The monocyte culture from normal individuals in the absence or the presence of rHuTGF-β1. *P < 0.05 vs. day 0; ^#^*P* < 0.05 as indicated. (mean ± SEM; *N* = 4). Monocyte-derived fibrocytes from normal individuals (*N* = 7) in the presence of rHuTGF-β1 (**c**) or TGF-βRI inhibitor SB431542 (**d**) in 3-day cultures. Horizontal line indicates the mean value. *P < 0.05. (**e**) Immunofluorescence analysis of fibrocyte culture as indicated in c and d. Arrows indicate fibrocytes stained with DAPI (blue), Phalloidin (green) and collagen I (red).

To investigate the direct impact of TGF-β1 on the differentiation of monocytes to macrophages, CD14^+^ monocytes from healthy donors and asthmatics were treated with recombinant human granulocyte macrophage colony-stimulating factor (rHuGM-CSF) (100 ng/ml) for 3 days with or without the addition of recombinant human TGF-β1 (rHuTGF-β1) (25 pg/ml). During the differentiating process of monocytes to macrophages, the percentage of PM-2K^+^ cells was increased significantly from day 1 to day 3 as determined by flow cytometry, but the level of enhancement was significantly lower in monocyte cultures from asthmatics than those from normal controls (Fig. 6a). As the increased levels of TGF-β1 were noted in monocytes of asthmatics, we next investigated the role of exogenous TGF-β1 in the generation of PM-2K^+^ cells and fibrocytes from CD14^+^ monocytes. The results showed that the percentage of the PM-2K^+^ cells from normal controls was significantly reduced by the addition of rHuTGF-β1 (Fig. 6b) over the three-day culture period, while, in contrast, the numbers of fibrocytes were significantly increased, in a dose-dependent manner (Fig. 6c), as compared with that of the medium control. Also, in the presence of a TGF-βRI inhibitor, SB431542, the generation of fibrocytes in culture could be dose-dependently inhibited (Fig. 6d). Moreover, immunofluorescence analysis clearly identified spindle-shaped fibrocytes with collagen I expression and oval nucleus, particularly when rHuTGF-β1 was present in culture (Fig. 6e). These data suggested that TGF-β1 signaling inhibited monocyte differentiation to PM-2K^+^ macrophage-like cells, but promoted fibrocyte differentiation *in vitro*.

## Discussion

We described herein an altered pattern of circulating monocyte differentiation in subjects with asthma, which was particularly noted in a group of subjects with severe asthma. While the molecular nature of the PM-2K marker remains to be elucidated, it has been used to distinguish the macrophage population in lung tissue^13^, in PBMCs from Kawasaki disease^15^, and derived from monocyte *in vitro*^12^. In the present study, a flow cytometric protocol was developed for identifying circulating PM-2K^+^ macrophage-like cells and distinguishing them from fibrocytes with appropriate FMO controls and elimination of doubling cells. Our results showed that the percentages of macrophage-like subsets (PM-2K^+^CD14^+^ and PM-2K^+^CD14^−^) were found to be significantly lower in subjects with asthma when compared to those of normal controls. Interestingly, when the subset of macrophage-like cells was further divided into two cell populations based on CCR7 and CD86 markers, significantly higher percentage of the CCR7^+^CD86^+^ and lower frequency of the CCR7^−^CD86^+^ subsets were found in asthmatic patients, correlating with decreased lung function.

In the present study, we identified a novel PM-2K^+^ subset with lower forward- and side-scatter patterns within the “small cell” gate shown in a flow cytometry plot (Fig. 2a) and observed the relationship between this subset and fibrocytes in asthmatic patients. In fact, PM-2K marker constitutively expressed in CD14^+^ monocytes as shown in Fig. 2e, Fig. 6a, and Supplementary Fig. S2. While the nature of this PM-2K^+^ cell population within the “small cell” gate or within the monocyte population is, at present, unclear, it could reflect different stages of cell activation and/or differentiation in circulation. Further detailed investigation is clearly needed to reveal the identity of this population.

Of interest to note, an inverse correlation between the frequencies of fibrocytes and PM-2K^+^ macrophage-like cells was evident only in the control group, but not in the patient group. In fact, in the patient group, significantly higher levels of fibrocytes were found, concomitant with significantly increased monocyte-derived TGF-β1 in asthmatic subjects. This was consistent with our findings that TGF-β1 was shown to inhibit the generation of PM-2K^+^ macrophage-like cells, while promoting the fibrocyte differentiation, and that the TGF-β1 level was significantly associated with the severity of asthma and its control status. As a corollary, while the exact role of fibrocytes in asthma remains to be fully defined, in a previous study^14^ circulating fibrocytes are reported to have increased percentage in patients with chronic airway obstruction, while the serum levels of TGF-β1 were significantly higher in a group of ten patients with chronic obstructive asthma than in normal subjects and in asthmatic cases with normal lung function. It is thus likely that TGF-β1 production by monocytes may play a role, in an autocrine mechanism, in controlling the differentiation of fibrocytes and macrophages. The collective evidence may lend a strong support for the existence of a dynamic change in monocyte differentiation associated with elevated levels of TGF-β1 in asthma.

It is noted that while, like circulating dendritic cells, the frequency of circulating PM-2K^+^ macrophage-like cells was relatively low, this population could be routinely discerned in the peripheral blood and monocyte-derived TGF-β1 appeared to be a negative regulator. It is, at present, unclear as to whether the seemingly systemic change in macrophage activation/differentiation pattern reflects what have occurred locally at sites of pulmonary mucosa. It is tempting to speculate that the decrease in the percentage of circulating PM-2K^+^ macrophage-like cells, particularly the CCR7^−^CD86^+^ subset, might indicate an active influx of circulating macrophage into the tissue, which may, in conjunction with eosinophil-derived TGF-β1^16^ at site of tissue mucosa, regulate pulmonary function, severity and control status. In fact TGF-β1-driven lung fibrosis has been shown to be macrophage dependent^17^. Further investigation into this hypothesis is clearly needed, particularly in regard to its functional importance. Moreover, the potential drug effect cannot be completely ruled out at this time, as the patients’ medication was not stopped prior to the outpatient visit. Further controlled study in an expanded study population is needed to properly address this issue.

Currently, there is still a lack of clinically efficacious biomarkers in defining asthma and for monitoring the disease activity, progression, severity and therapeutic outcomes. One exception, perhaps, is the fraction of exhaled NO (FeNO), which has been used to distinguish asthma from non-asthma^18,19^. But, it is a relatively non-specific marker of response and has, thus far, no value in defining a more specific disease phenotype and its severity^20^.

Our current study with a relatively larger sample size suggest that the use of multi-color flow cytometry with a small amount of peripheral blood could detect circulating monocytes/macrophages and their differentiation status, supporting the potential utility of PM-2K and antigen presenting cell-associated markers in discriminating severe asthma, which may offer a new approach in our understanding of asthma and a potentially novel target for investigating the pathogenic mechanisms of asthma. Also, in the present study, not only subjective parameters (ACT scores), but also the levels of lung functions and severity were correlated with these markers (PM-2K, CD14, CCR7, CD86). These quantitative data may serve as a basis for analysis of an expanded study population for testing its clinical utility. Further, this analytical platform may be generally applicable in the study of various diseases where macrophages are known to be important in the pathogenesis or resolution of diseases.

## Methods

### Study population

The study population included, prospectively, adult asthma patients at the outpatient departments of seven hospitals in southern Taiwan. Patients who met the following inclusion criteria were eligible for enrollment: (1) at least 18 years of age, (2) physician-diagnosed asthma. Asthma patients with concomitant pulmonary diseases, including pulmonary tuberculosis, pulmonary tumors, fibrotic cysts, chronic bronchitis, emphysema, and bronchiectasis were excluded. Physician’s diagnosis of asthma and its severity were made according to an operational description suggested by the Global Initiative for Asthma guidelines^21^. The severity of asthma was classified on the basis of the intensity of treatment required to achieve good asthma control. As suggested by the GINA guidelines, severe asthma is asthma that requires high intensity treatment, e.g. GINA step 4 or 5, to maintain good control, or where good control is not achieved despite high intensity treatment. In this study, we further stratified asthma into four groups: asthma that required GINA step 1 or 2 to maintain good control was considered as mild asthma, GINA step 3 as moderate, GINA step 4 as severe, and GINA step 5 as very severe.

Patients with severe asthma (step 4) were receiving more than two combination controller therapy (inhaled corticosteroids, long-acting β-adrenoceptor agonist, leukotriene modifier and sustained-release theophylline), while step 5 asthmatics were treated with oral corticosteroid or anti-IgE Abs, in addition to step 4 therapy. Patients were also evaluated for their control status by using ACT, a validated patient-completed questionnaire consisting of five parameters aimed at assessing asthma symptoms (daytime and nocturnal), use of rescue medications, and the effect of asthma on daily functioning. The scores range from 5 (poor control of asthma) to 25 (complete control of asthma)^22^. The scores equal or less than 19 was considered to be “not well controlled”.

Pulmonary function was measured with a Jaeger Master screen Pulmonary System spirometer (Hoechberg, Germany). FEV_1_% or FVC% was expressed as percentages of the predicted values, whereas FEV_1_%/FVC% was defined as FEV_1_ of predicted value divided by FVC of predicted value. Decline of FEV_1_% was defined as one minus FEV_1_ of predicted value. If the value of decline of FEV_1_% was below zero, it was considered as zero. The healthy volunteers, who underwent routine annual physical examination, had normal lung function and no history of asthma, were also included for comparison. The study protocol was approved by the Institutional Review Boards of Kaohsiung Medical University Hospital (KMUH-IRB-990392). After informed consent was obtained, peripheral blood samples were obtained from healthy individuals and asthmatic patients. Case-control comparisons were implemented, depending on the availability of the respective samples at the time of analysis. The study was performed in accordance with the ethical standards laid down in the 1964 Declaration of Helsinki and its later amendments.

### Flow cytometry analysis

For simultaneously staining PM-2K^+^ cells and fibrocytes, fluorochrome-conjugated monoclonal antibodies against the following antigens were used: CD3, CD14, CD19, CD45RO, collagen I and a specific marker of macrophages, PM-2K^12^. Ficoll-isolated PBMCs (Ficoll-Paque Plus, GE Healthcare, Biosciences, Piscataway, NJ) were sequentially stained with human Fc receptor binding inhibitor (eBioscience), purified anti-macrophage Abs (PM-2K, Serotec) and followed by anti-mouse IgG-FITC. After washing, the cells were then fixed and stained intracellularly with rabbit IgG anti-collagen I (Rockland) and anti-rabbit IgG-Qdot655 (Invitrogen) using intracellular staining kit (eBioscience). After washing, the cells were then stained with other fluorochrome-labeled monoclonal antibodies against surface markers in PBS containing 0.5% fetal bovine serum, including CD3-Pacific blue (UCHT1, BD), CD19-Pacific blue (HIB19, eBioscience), CD14-PE/Cy7 (61D3, eBioscience), and CD45RO-PE (UCHL1, BD) and appropriate isotype controls. For analysis of PM-2K^+^ subsets, PBMCs were stained with PM-2K antibody, followed by anti-mouse IgG-FITC. After washing, cells were then stained with fluorochrome-conjugated monoclonal antibodies against surface markers, including CD3, CD19, CD14, CD86 and CCR7.

FMO controls specific for PM-2K, collagen I, CD86, or CCR7 markers for each sample were used to facilitating recognition of boundaries between positive and negative subsets. For example, to determine the positive boundary for collagen I expression in CD3^−^CD19^−^CD45^+^ cells (Fig. 1a), a FMO control was prepared in which cells were stained with all fluorochrome-conjugated monoclonal antibodies except the one that recognizes collagen I^23^. The samples were run on a LSRII flow cytometer (BD Biosciences, San Jose, CA) with data acquisition on 10^6^ live cells (gated by forward and side scatter properties) and analyzed using FlowJo software (Tree Star, Inc, Ashland, OR). Moreover, the detailed technical protocols regarding the blocking step after the anti-mouse IgG-FITC staining and the fixation effect on staining pattern are provided in Supporting Information with pertinent data shown in Supplementary Fig. S3, Figs. S4 and S5, respectively.

### *In vitro* analysis of monocyte-derived PM-2K^+^ cells and fibrocytes

Purified CD14^+^ cells (90%–95% of purity) from PBMCs were positively selected using CD14 microbeads (Miltenyi Biotec, Bergisch Gladbach, Germany) according the manufacturer’s instruction. For the analysis of monocyte-derived PM-2K^+^ cells *in vitro*, purified CD14^+^ monocytes from healthy donors and asthmatic subjects were cultured for 3 days in the presence of 100 ng/ml rHuGM-CSF (R&D Systems, Minneapolis, MN)^24^. The levels of TGF-β1 in the supernatants of CD14^+^ monocytes cultured for 24 hours without any stimulation were analyzed by enzyme-linked immunosorbent assay (R&D Systems). In some cases, 25 pg/ml of rHuTGF-β1 (R&D Systems) was added and its effect on the frequency of PM-2K^+^ cells was analyzed by flow cytometry.

For *in vitro* fibrocyte culture, CD14^+^ monocytes were cultured in FibroLife basal media (Lifeline Cell Technology, Walkersville, MD) as described previously^25,26^, in the presence or absence of a TGF-βRI inhibitor, SB431542^27^ (Sigma-Aldrich Co., St. Louis, MO), or rHuTGF-β1 for 4 days and counted with the use of AxioVision 4.8 software. Fibrocytes were defined as spindle-shaped cells with an oval nucleus, as described previously^10,25^.

### Statistical analysis

Statistical analysis was performed using IBM SPSS Statistics (Version 19, IBM Company, Armonk, NY, USA) and GraphPad Prism (Version 5, GraphPad Prism Software, Los Angeles, CA, USA). Mann-Whitney U test was used to determine the difference between normal subjects and patients. Kruskal-Wallis test with post-hoc Dunn’s multiple comparison test was used to determine the difference between subgroups of patients. Spearman correlation test was used to determine the correlation between the indicated subsets and ACT scores as well as other clinical parameters (severity and lung function tests). *P* value < 0.05 was considered significant.

## Electronic supplementary material

Supporting information
