# Supplementary material for: Altered pattern of monocyte differentiation and monocyte-derived TGF-β1 in severe asthma
Source: Sci Rep. 2018 Jan 17;8:919. doi: 10.1038/s41598-017-19105-z (PMC5772494; doi:10.1038/s41598-017-19105-z)
Supplement: Supplementary file 1 — Supporting information [file 41598_2017_19105_MOESM1_ESM.doc]

**Altered pattern of monocyte differentiation and monocyte-derived TGF-β1 in severe asthma**

Chih-Hsing Hung^¶^, Chin-Chou Wang^¶^, Jau-Ling Suen^¶^, Chau-Chyun Sheu, Chang-Hung Kuo, Wei-Ting Liao, Yi-Hsin Yang, Chao-Chien Wu, Sum-Yee Leung, Ruay-Sheng Lai, Chi-Cheng Lin, Yu-Feng Wei, Chong-Yeh Lee, Ming-Shyan Huang^&^ & Shau-Ku Huang^&^*

**Supplemental Methods**

*Flow cytometry analysis: blocking step after the anti-mouse IgG-FITC staining*

In order to avoid non-specifically binding the other mouse mAb by anti-mouse IgG-FITC as there is likely to be some of the secondary anti-mouse IgG FITC that only has one of the two IgG binding sites bound to the PM-2K mAb, different blocking buffers were tested after anti-mouse IgG-FITC staining. As shown in Supplementary Fig. S3, the staining buffers, 0.5% or 2% mouse serum in 1× PBS, were used to compare data from the original staining buffer (0.5% fetal bovine serum (FBS) in 1× PBS). The staining protocol was the same with that in Fig. 2 (without fixation treatment) in the manuscript, and was briefly described in the Supplementary Fig. S3 legend. The result showed that the staining profiles were very similar among these three conditions, including cell distribution in FSC versus SSC plot, the percentages of non-lymphocytes and of PM-2K^+^CD14^+^ cells, and CD86 versus CCR7 distribution pattern within PM-2K^+^CD14^+^ gate. Therefore, the results suggest that non-specific binding of antibodies is minimal, if at all, in our current Flow analysis.

*The fixation effect on staining pattern*

In order to understand whether the percentages of lymphocyte subsets were altered by fixation treatment and whether the CD19 mAb (cone HIB19) was sensitive to fixation treatment in the present study, FITC-anti-CD3 and Pacific blue-anti-CD19 were initially used to stain PBMCs from one healthy donor under non-fixation condition. As shown in the Supplementary Fig. S4A, the T cell (CD3^+^CD19^-^) percentage is 69.3% and B cell (CD3^-^CD19^+^) percentage is 11.4%, so the total lymphocyte percentage is around 80.7%. In the second set of experiment, Pacific blue-anti-CD3 and Pacific blue-anti-CD19 were used to stain PBMCs under non-fixation condition, which is similar to that shown in Fig. 2 in the manuscript. The data showed that CD3^+^CD19^+^ cells was 81.0% (Supplementary Fig. S4B). In the third set of experiment, Pacific blue-anti-CD3 and Pacific blue-anti-CD19 was used to stain PBMCs after fixation treatment, which is similar to that shown in Fig. 1 in the manuscript. As shown in Supplementary Fig. S4C, the percentage of CD3^+^CD19^+^ cells was still 81.0%, although the cell distribution was altered in FSC versus SSC plot after fixation treatment. Therefore, it seems that the binding epitope of CD19 was not lost in our staining protocol used in the present study.

In order to identify the subset within CD3^-^CD19^-^ small cells and show the effect of fixation protocol, PBMCs were stained with new panel of flourchrome-conjugated monoclonal antibodies with (Supplementary Fig. S5B) or without fixation (Supplementary Fig. S5A). The antibody panel and staining protocols were described in the legend of Supplementary Fig. S5 below. Similar to Fig. Supplementary Fig. S4, the percentages of CD3^+^ T cells and CD19^+^ B cells from non-fixation experiment were similar to those noted in fixation experiment (Supplementary Figs. S5A and S5B). Also, similar percentages (around 60%) of CD3^-^CD16/CD56^+^ NK cells within the CD3^-^CD19^-^ subset were observed in either non-fixation or fixation experiment. These data show that the major cell subset within the “CD3^-^CD19^-^ small cells” is NK cells, and the staining pattern was not significantly affected by fixation protocol in the present study.

**Supplemental figures**

**
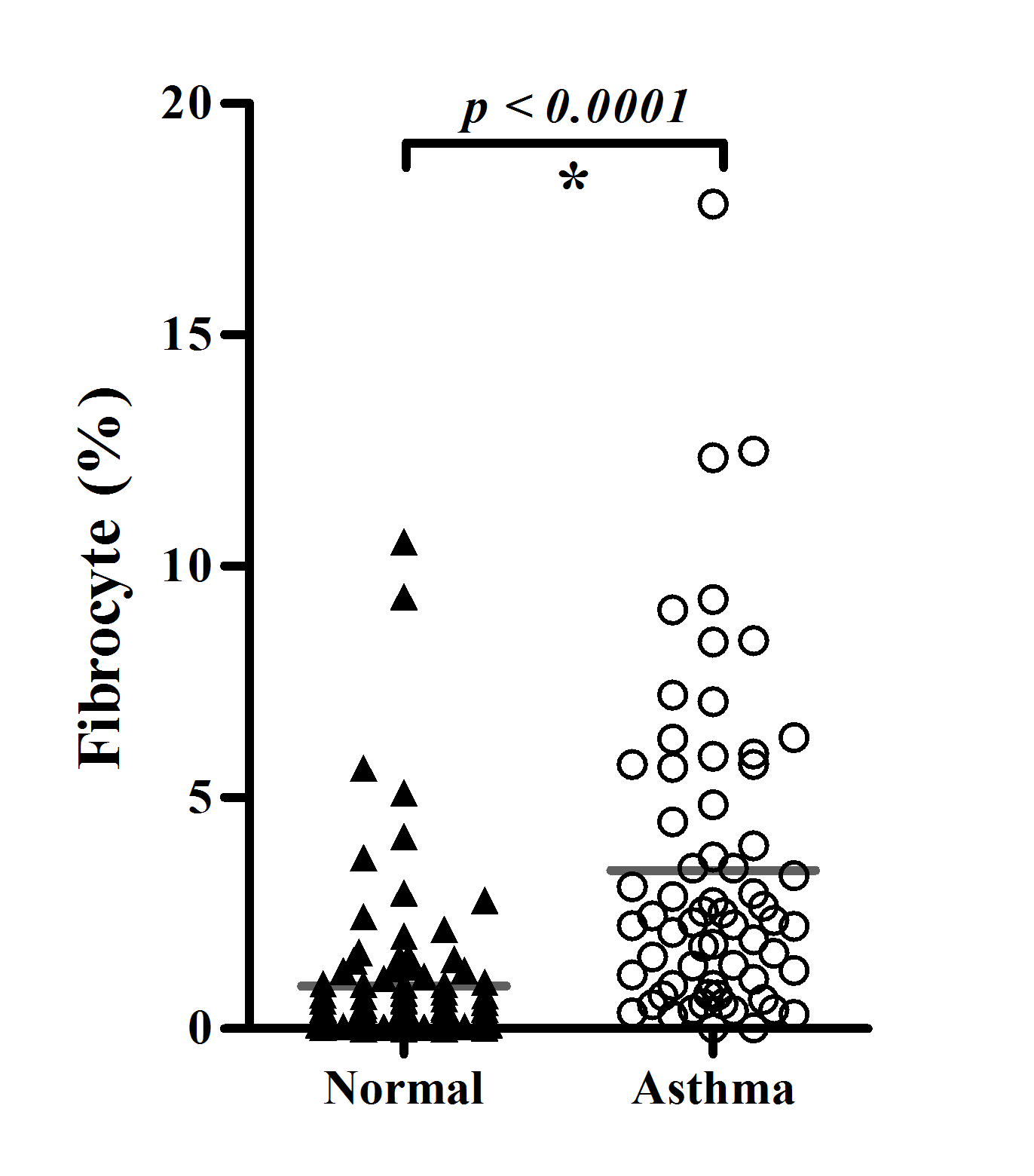
**

**Supplementary Fig. S1.** The percentage of fibrocytes in peripheral blood mononuclear cells. Normal, *N* = 97, Asthma, *N* = 64. The horizontal line marks the mean value. *: *P* < 0.05 was considered significant.


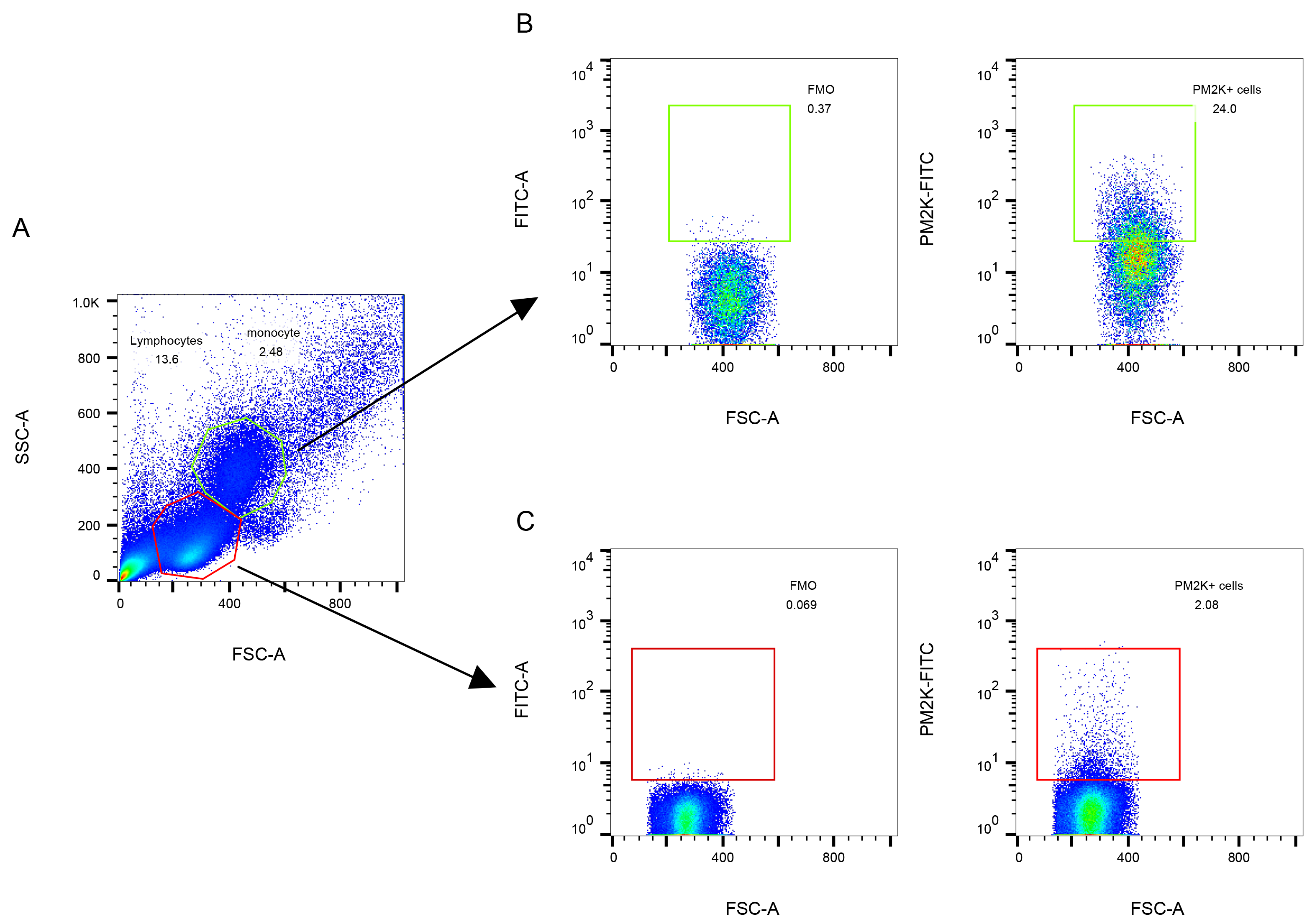


**Supplementary Fig. S2.** PM-2K^+^ cells in peripheral blood. Ficoll-isolated PBMCs from one normal individual were stained with PM-2K antibody, followed by FITC anti-mouse IgG. After washing, cells were then stained with Alexa700 anti-CD14. PM-2K expression was shown in the “monocyte” gate (green) (B) or “small cell” gate (red) (C). The boundary between PM-2K^+^ and PM-2K^-^ cells was set based on the fluorescence minus one control specific for PM-2K.


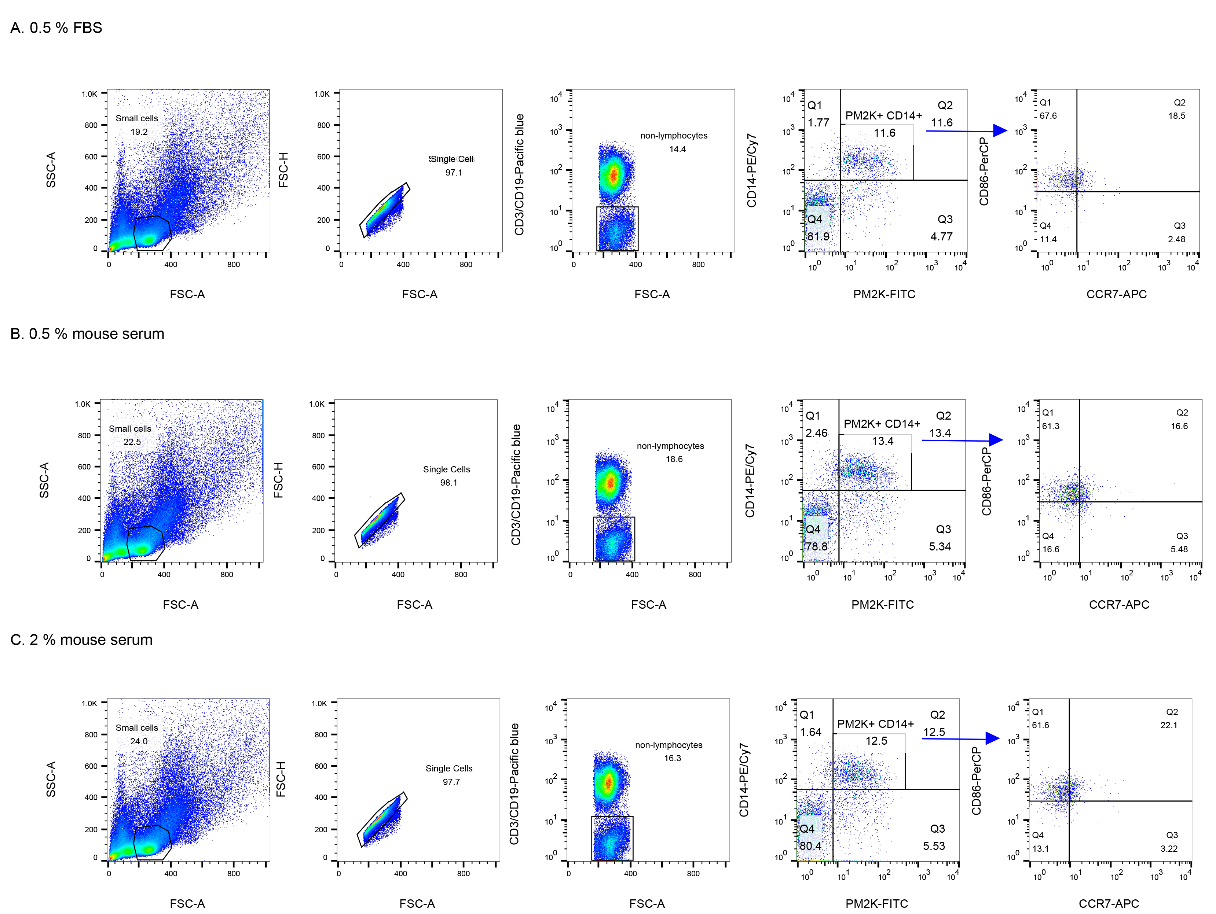


**Supplementary Fig. S3.** PM-2K staining pattern with murine Ig blocking step. PBMCs from one healthy donor were stained with human Fc receptor binding inhibitor, purified anti-macrophage Abs (PM-2K, Serotec) and followed by anti-mouse IgG-FITC. After washing, the cells were re-suspended in 1X PBS buffers containing either 0.5% fetal bovine serum (FBS; A), 0.5% mouse serum (B) or 2% mouse serum (C), and then stained with the fluorochrome-conjugated monoclonal antibodies against surface markers, including CD3, CD19, CD14, CD86 and CCR7.


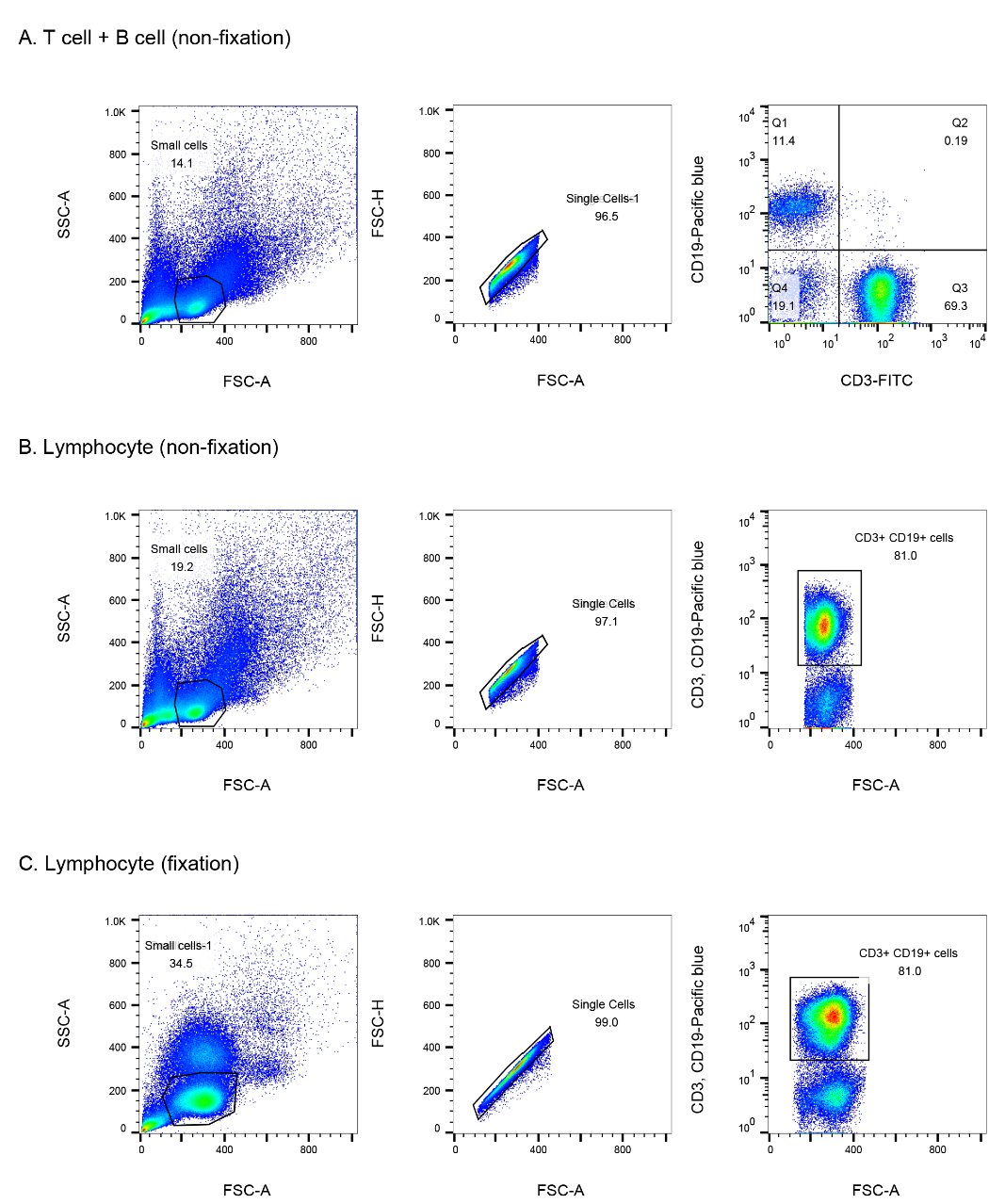


**Supplementary Fig. S4.** Lymphocyte percentages under fixation and non-fixation conditions. PBMCs from one healthy donor were stained with FITC-anti-CD3/Pacific blue-anti-CD19 (A), or Pacific blue-anti-CD3/Pacific blue-anti-CD19 (B) without fixation treatment. (C) PBMCs were fixed (eBioscience; 00-8222), permeabilized and washed (eBioscience; 00-8333) according the manufacturer’s instruction. After washing, the cells were then stained with Pacific blue-anti-CD3/Pacific blue-anti-CD19. Both FITC-anti-CD3 and Pacific blue-anti-CD3 antibodies are clone UCHT1; while Pacific blue-anti-CD19 antibody is clone HIB19.


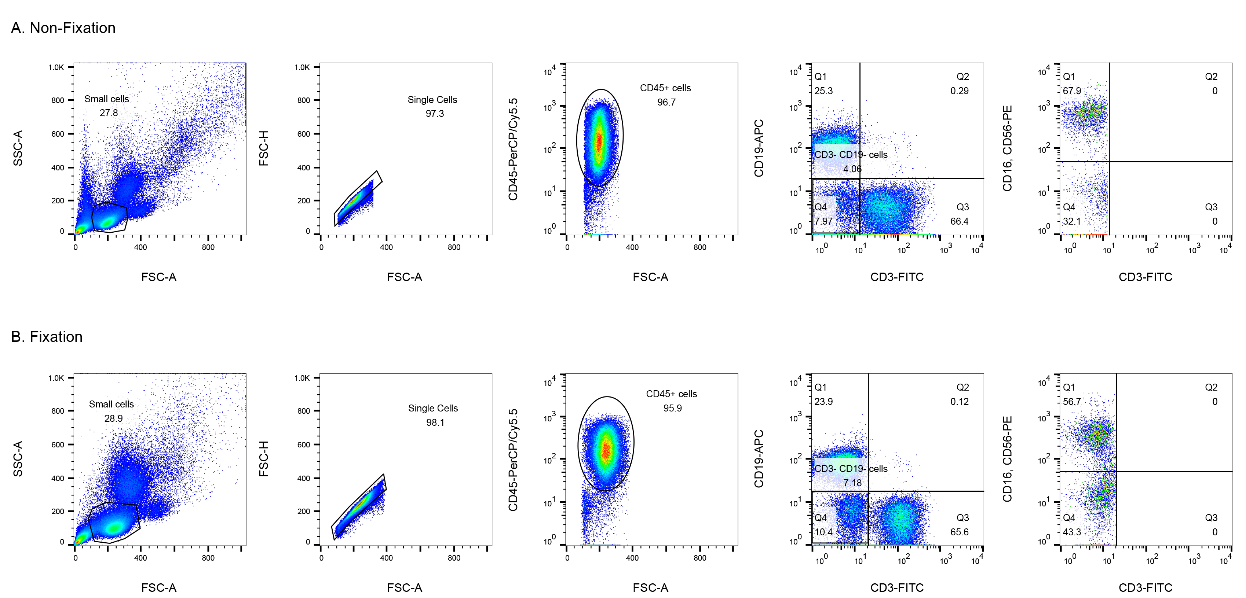


**Supplementary Fig. S5.** Identification of NK cells under fixation and non-fixation conditions. (A) PBMCs were stained with flourchrome-conjugated monoclonal antibodies under non-fixation protocol. The antibody panel includes FITC-anti-CD3, APC-anti-CD19, PercP/Cy5.5-anti-CD45 (2D1), PE-anti-CD16 (B73.1) and PE-anti-CD56 (NCAM16.2). (B) PBMCs were fixed (eBioscience; 00-8222), permeabilized and washed (eBioscience; 00-8333) according the manufacturer’s instruction. After washing, the cells were then stained with the antibody panel as described in A.

**Supplemental tables**

**Supplementary Table S1.** ROC analysis of the CCR7^-^CD86^+^ subset in distinguishing the severe/very severe asthma group (*N* = 53)

| Variable | AUC | Cutoff point | Sensitivity | Specificity |
| --- | --- | --- | --- | --- |
| CCR7^-^CD86^+^ (%)  in PM-2K^+^CD14^+^ | 0.68 | 41.27 | 0.62 | 0.75 |
| CCR7^-^CD86^+^ (%)  in PM-2K^+^CD14^-^ | 0.68 | 6.65 | 0.76 | 0.60 |

**Supplementary Table S2. Cytokine and chemokine pattern secreted from CD14^+^ monocytes from asthma patients and healthy controls**

| (pg/ml) | Normal (*N* = 128)  Median (25, 75 percentile) | Asthma (*N* = 109)  Median (25, 75 percentile) | *P* value |
| --- | --- | --- | --- |
| TGF-β1 | 44.7 (34.4, 75.1) | 98.4 (43.9, 482.2) | *** *P* < 0.0001** |
| IL-10 | 0 (0, 102.3) | 10.9 (0, 130.5) | *P* = 0.5033 |
| TNF-α | 290.9 (37.7, 737.2) | 132.7 (26.4, 555.2) | *P* = 0.2917 |
| CCL1  (I-309) | 190.1 (80.8, 426.2) | 0 (0, 108.7) | *** *P* < 0.0001** |
| CCL2  (MCP-1) | 278.3 (40.0, 1,236.0) | 26.2 (0, 440.8) | *** *P* = 0.0015** |
| CCL16 | 4.9 (0, 6.3) | 0 (0, 5.2) | *** *P* = 0.0022** |
| CCL17  (TARC) | 9.5 (6.5, 12.4) | 0 (0, 9.0) | *** *P* < 0.0001** |
| CCL22  (MDC) | 42.3 (20.1, 144.5) | 0 (0, 58.5) | *** *P* < 0.0001** |
| CXCL10  (IP-10) | 31.1 (18.7, 110.1) | 0 (0, 32.3) | *** *P* < 0.0001** |

* *P* value < 0.05 was considered significant.
